# Supplementary material for: Comparative Transcriptome Profiling Analysis Uncovers Novel Heterosis-Related Candidate Genes Associated with Muscular Endurance in Mules
Source: Animals (Basel). 2020 Jun 4;10(6):980. doi: 10.3390/ani10060980 (PMC7341310; doi:10.3390/ani10060980)
Supplement: Supplementary file 1 [file animals-10-00980-s001.pdf]

Supporting information

Additional supporting information may be found online in the supporting information tab for this article:

Table S1 Number of pathways enriched by the DEGs and DAS Genes.

Table S2 Table S2-1.xlsx**:** Pathways enriched in muscle, brain and skin tissues by DEGs and DAS genes that identified between mule and horse.

Table S3 Table S3-1.xlsx: Pathways in muscle, brain and skin tissues by DEGs and DAS genes that identified between mule and donkey.

Figure S1 The PCA of the brain, muscle, and skin tissues in horses, donkeys, mules and hinnies.

Figure S2 The venn diagrams of differentially expressed genes among three tissues.

Figure S3 Top 10 pathways enriched in muscle tissue by DEGs.

Figure S4 Top 10 pathways enriched in brain tissue by DEGs.

Figure S5 Top 10 pathways enriched in skin tissue by DEGs.

Figure S6 The venn diagrams of differentially expressed genes and differentially spliced genes in brain, muscle, and skin tissues.

Figure S7 Proportion of five splicing types of the DAS genes in three tissues.

Figure S8 Top 10 pathways enriched in muscle tissue by DAS genes.

Figure S9 Top 10 pathways enriched in brain tissue by DAS genes.

Figure S10 Top 10 pathways enriched in skin tissue by DAS genes.

Figure S11 Validation of DAS genes identified in the muscle tissues between mule and horse by the full-length transcriptome data generated from Pac-Bio sequencing platform.

Figure S12 The result of transcriptome data mapping on the *TNNC2* gene among the muscle tissues of hinny, mule, donkey and horse.

Figure S13 The result of transcriptome data mapping on the *RYR1* gene among the muscle tissues of hinny, mule, donkey and horse.

Table S1 Number of pathways enriched by the DEGs and DAS Genes.

Type	Tissues	Mules vs horses	Mules vs donkeys	Hinnies vs horses	Hinnies vs horses
DEGs	Muscle	1,030	84	778	979
	Brain	900	294	665	974
	Skin	1,045	458	798	850
DAS	Muscle	564	79	503	621
genes	Brain	377	108	358	484
	Skin	289	85	381	341

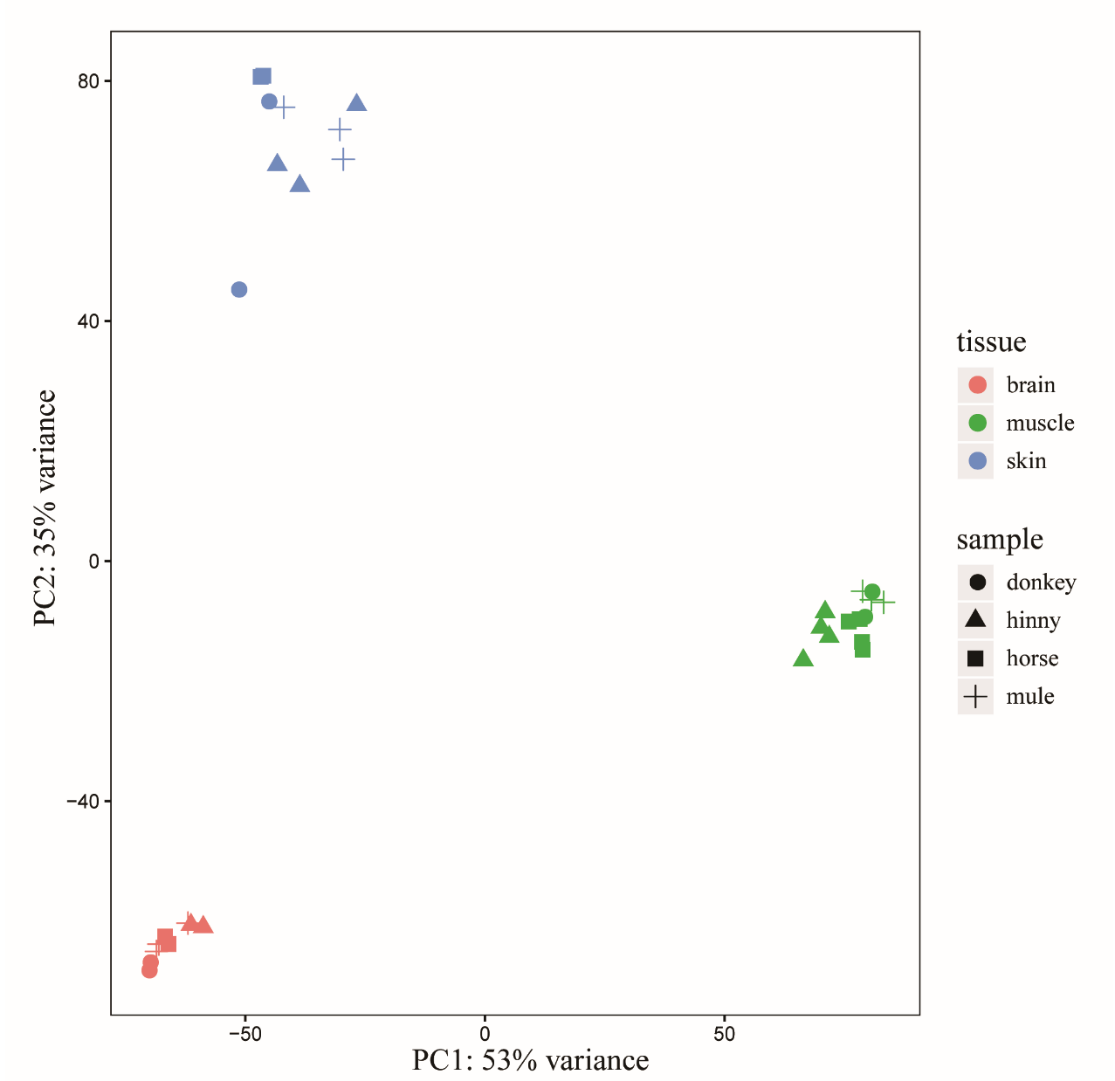

Figure S1 The PCA of the brain, muscle, and skin tissues in horses, donkeys, mules and hinnies.

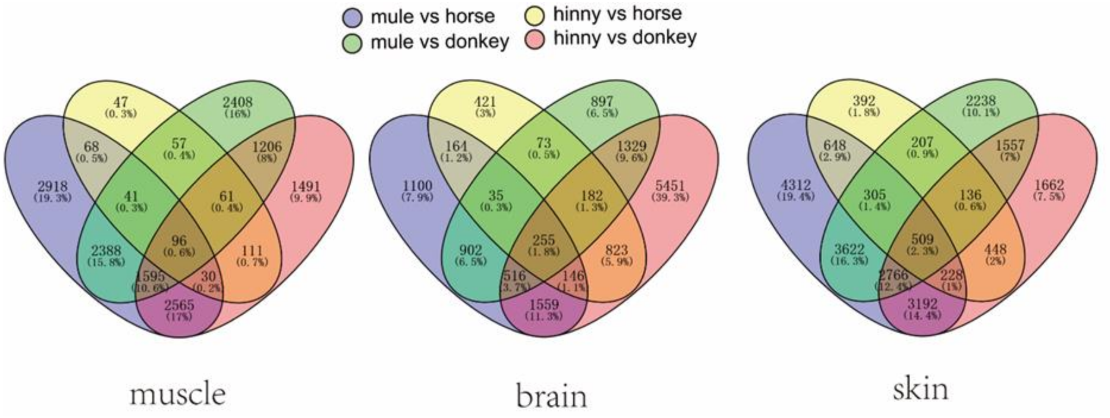

Figure S2 The venn diagrams of differentially expressed genes among three tissues.

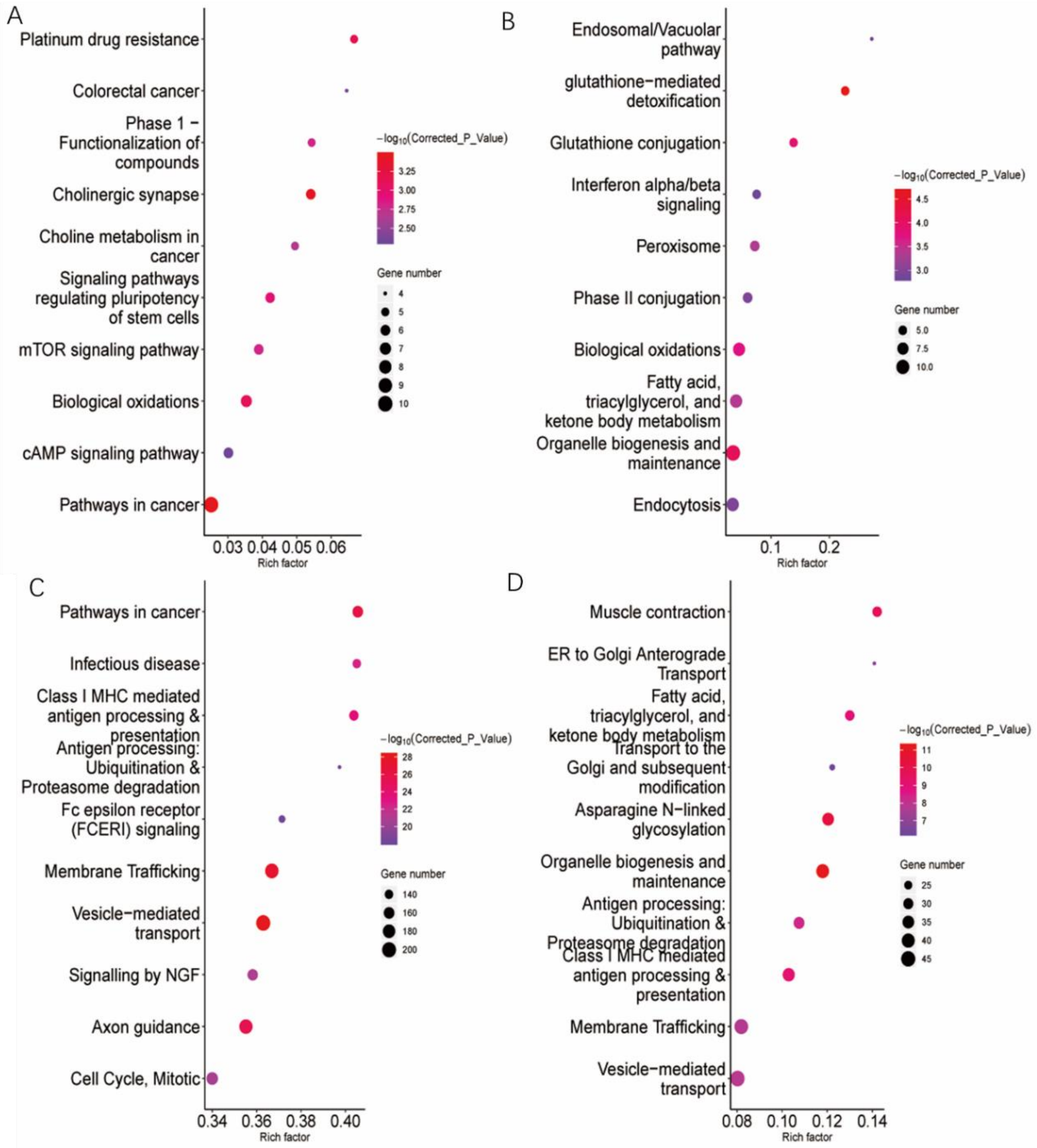

Figure S3 Top 10 pathways enriched in muscle tissue by DEGs. **A,** pathways enriched by the DEGs between mule vs donkey. **B,** pathways enriched by the DEGs between mule vs horse. **C,** pathways enriched by the DEGs between hinny vs donkey. **D,** pathways enriched by the DEGs between hinny vs horse.

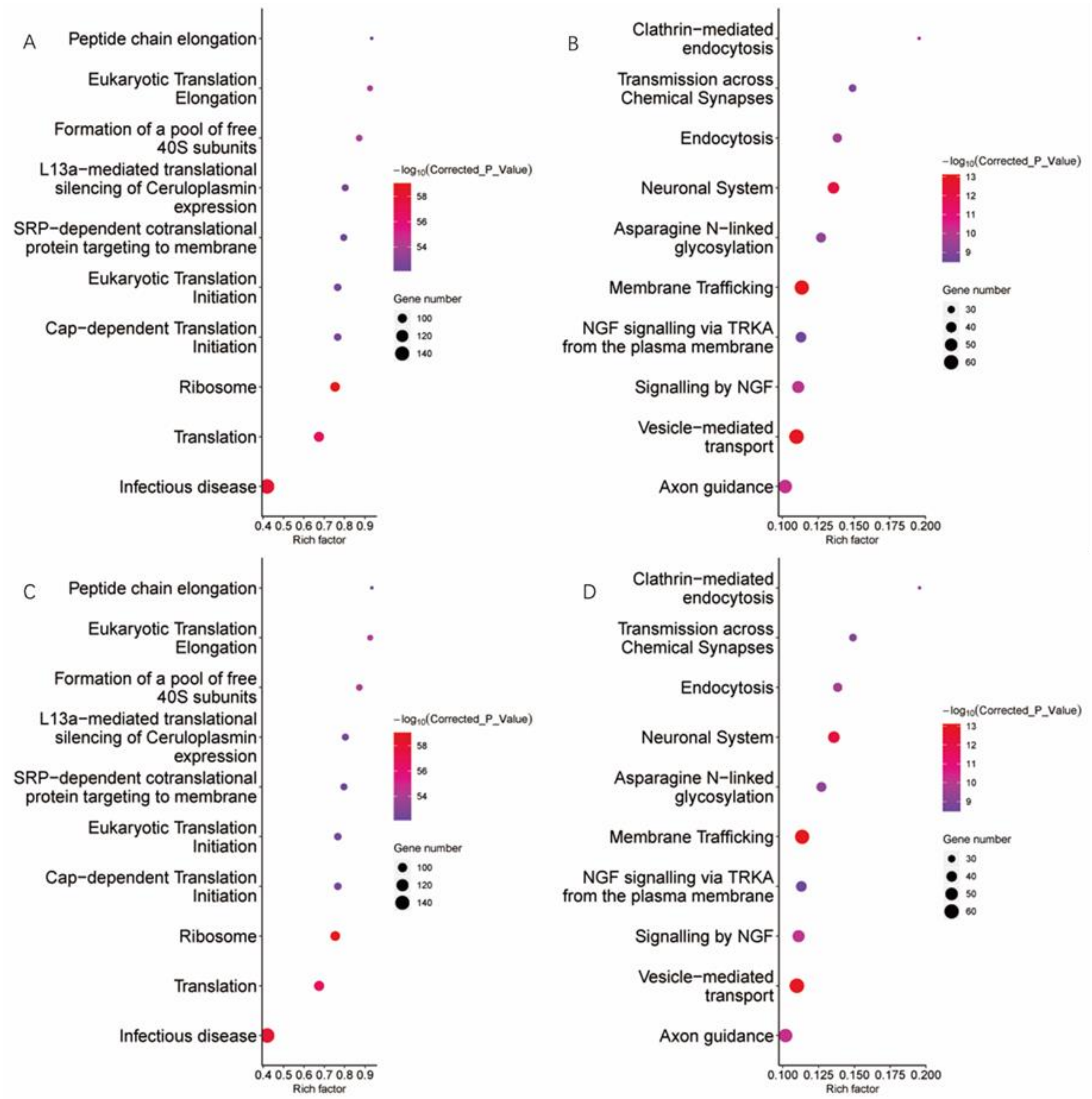

Figure S4 Top 10 pathways enriched in brain tissue by DEGs. **A,** pathways enriched by the DEGs between mule vs donkey. **B,** pathways enriched by the DEGs between mule vs horse. **C,** pathways enriched by the DEGs between hinny vs donkey. **D,** pathways enriched by the DEGs between hinny vs horse.

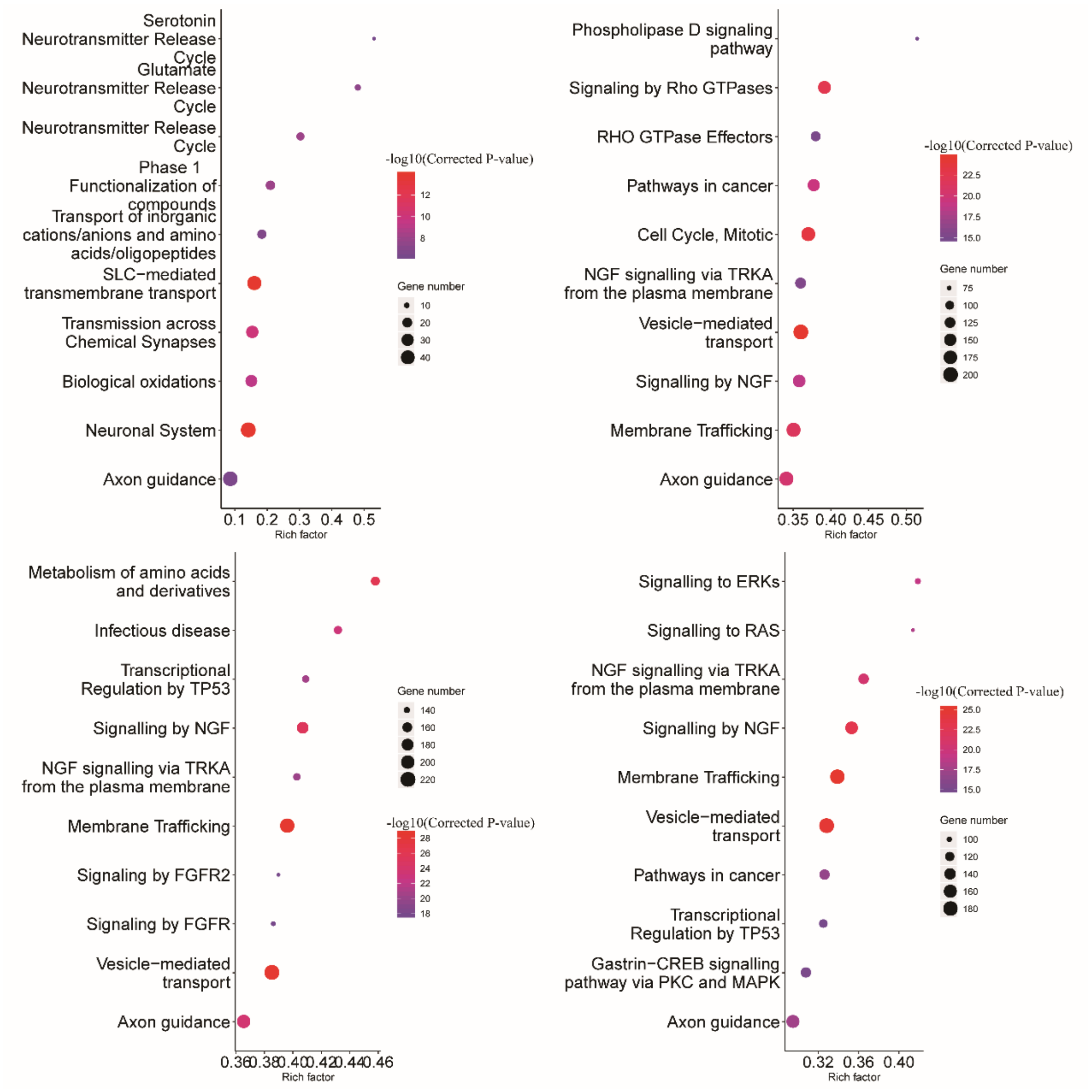

Figure S5 Top 10 pathways enriched in skin tissue by DEGs. **A,** pathways enriched by the DEGs between mule vs donkey. **B,** pathways enriched by the DEGs between mule vs horse. **C,** pathways enriched by the DEGs between hinny vs donkey. **D,** pathways enriched by the DEGs between hinny vs horse.

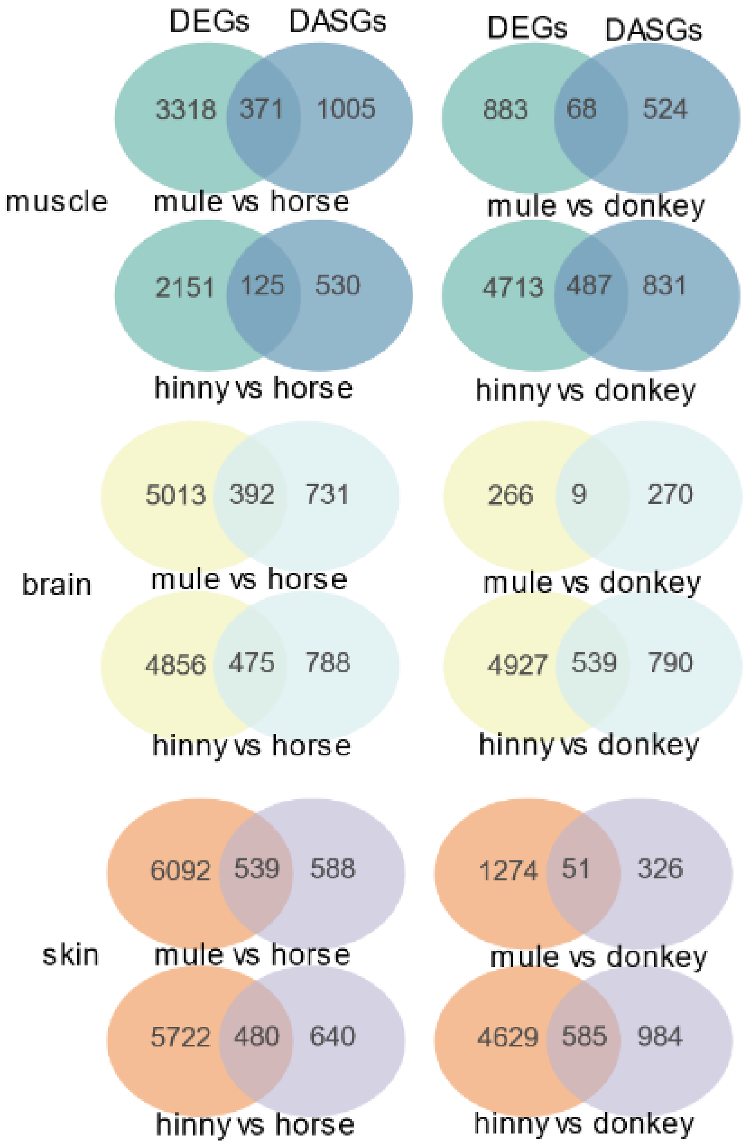

Figure S6 The venn of differentially expressed genes and differentially spliced genes in brain, muscle, and skin tissues.

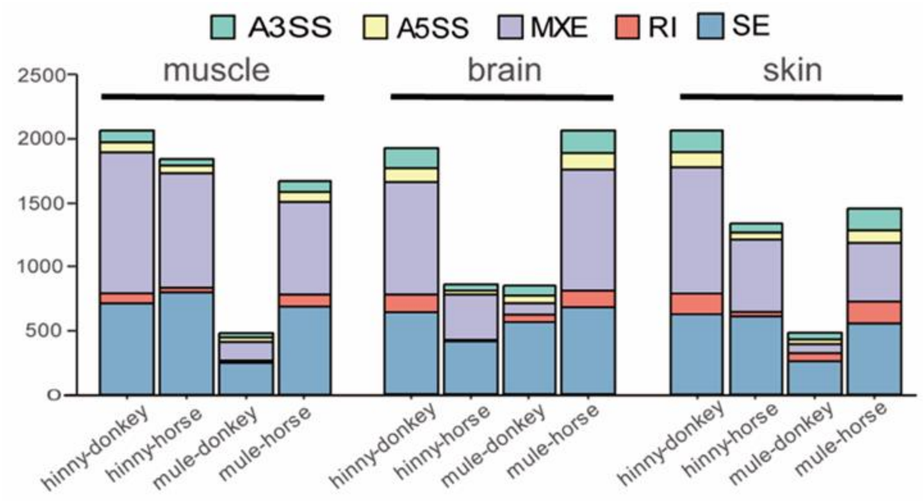

Figure S7 Proportion of five splicing types of the DAS genes in three tissues. A3SS, Alternative acceptor site **A5SS,** Alternative donor site **MXE,** Mutually exclusive exons **RI,** Intron retention **SE,** Exon skipping.

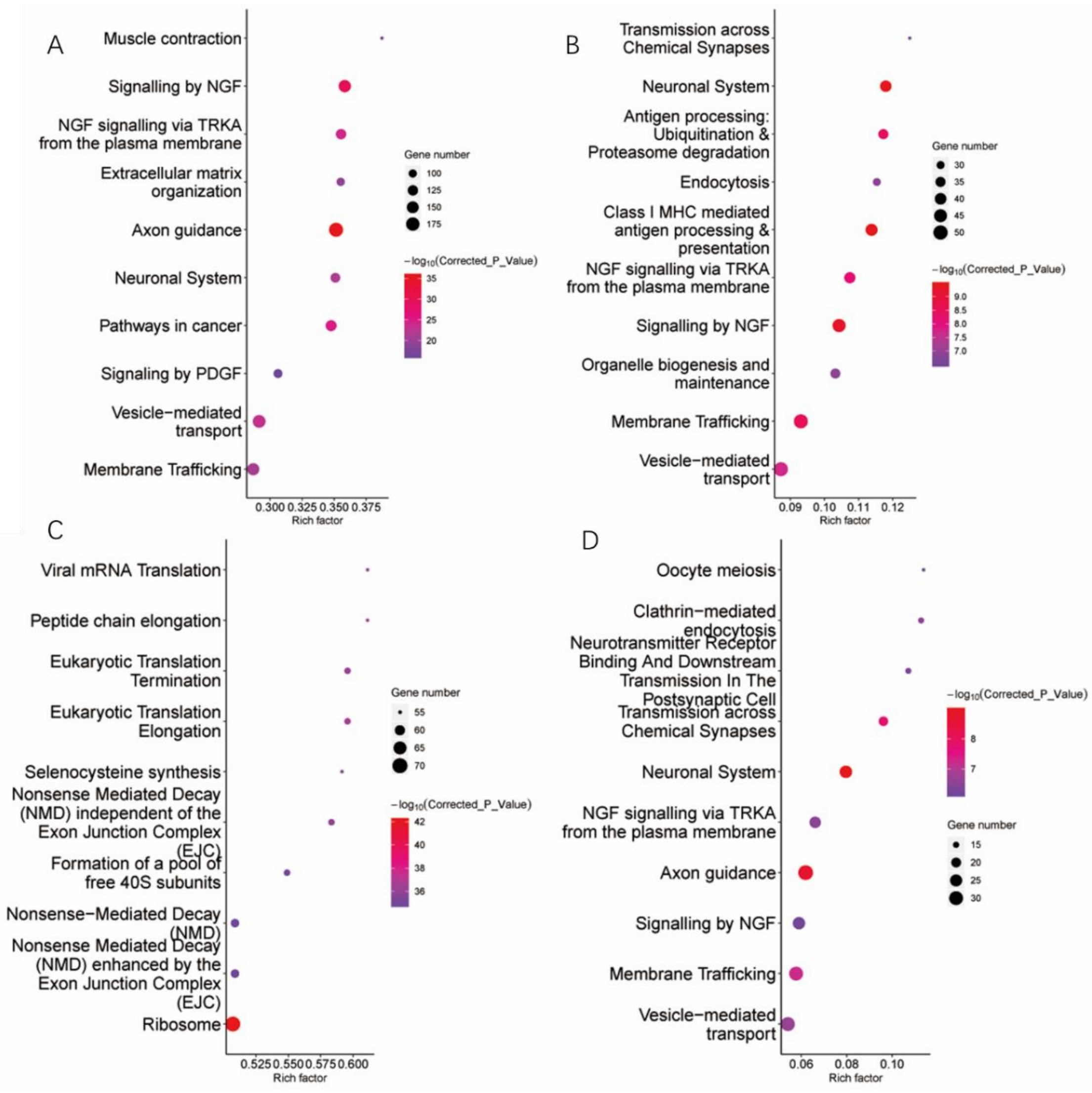

Figure S8 Top 10 pathways enriched in muscle tissue by DAS genes. **A,** pathways enriched by the DAS genes between mule vs donkey. **B,** pathways enriched by the DAS genes between mule vs horse. **C,** pathways enriched by the DAS genes between hinny vs donkey. **D,** pathways enriched by the DAS genes between hinny vs horse.

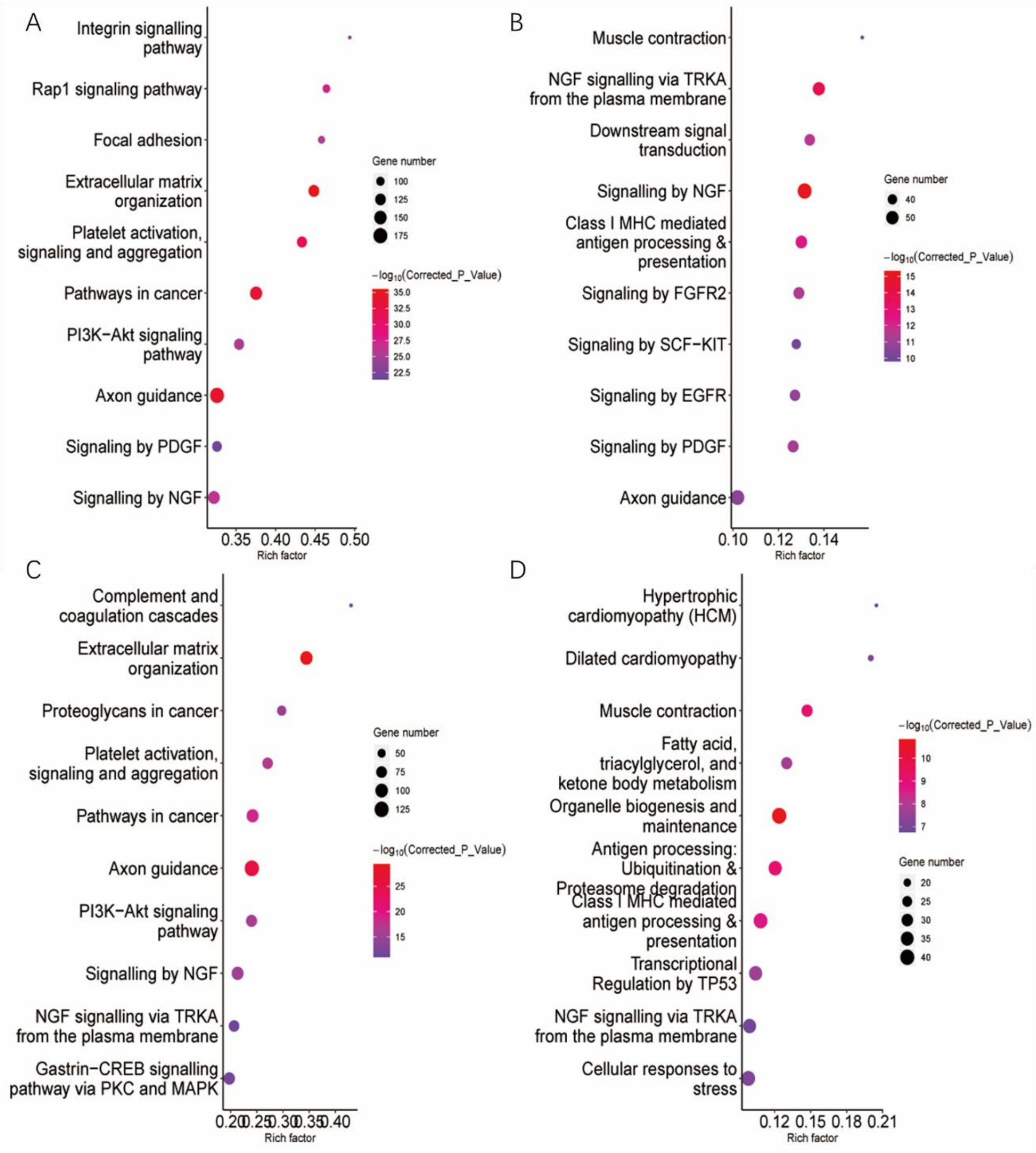

Figure S9 Top 10 pathways enriched in brain tissue by DAS genes. **A,** pathways enriched by the DAS genes between mule vs donkey. **B,** pathways enriched by the DAS genes between mule vs horse. **C,** pathways enriched by the DAS genes between hinny vs donkey. **D,** pathways enriched by the DAS genes between hinny vs horse.

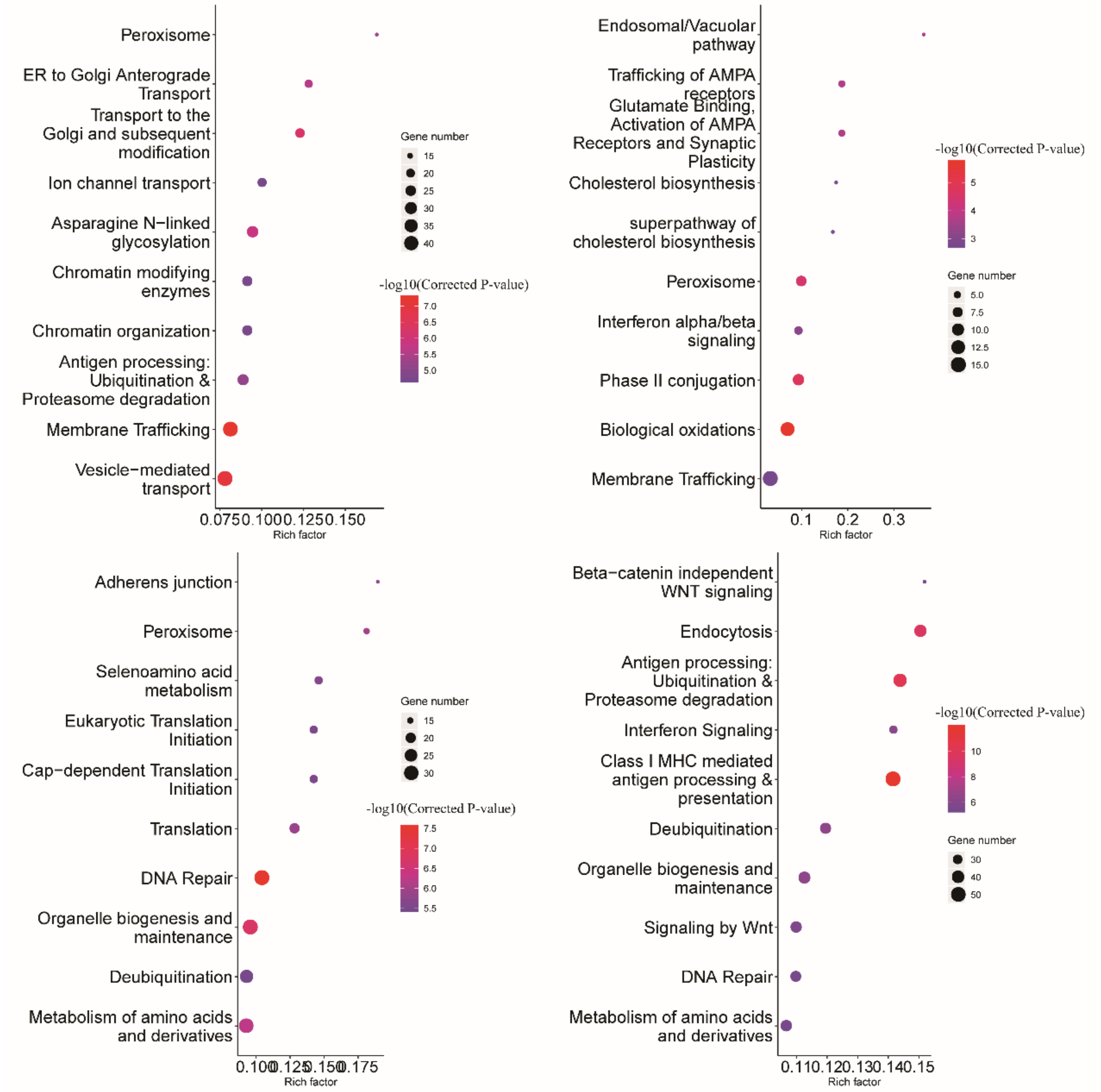

Figure S10 Top 10 pathways enriched in skin tissue by DAS genes. **A,** pathways enriched by the DAS genes between mule vs donkey. **B,** pathways enriched by the DAS genes between mule vs horse. **C,** pathways enriched by the DAS genes between hinny vs donkey. **D,** pathways enriched by the DAS genes between hinny vs horse.

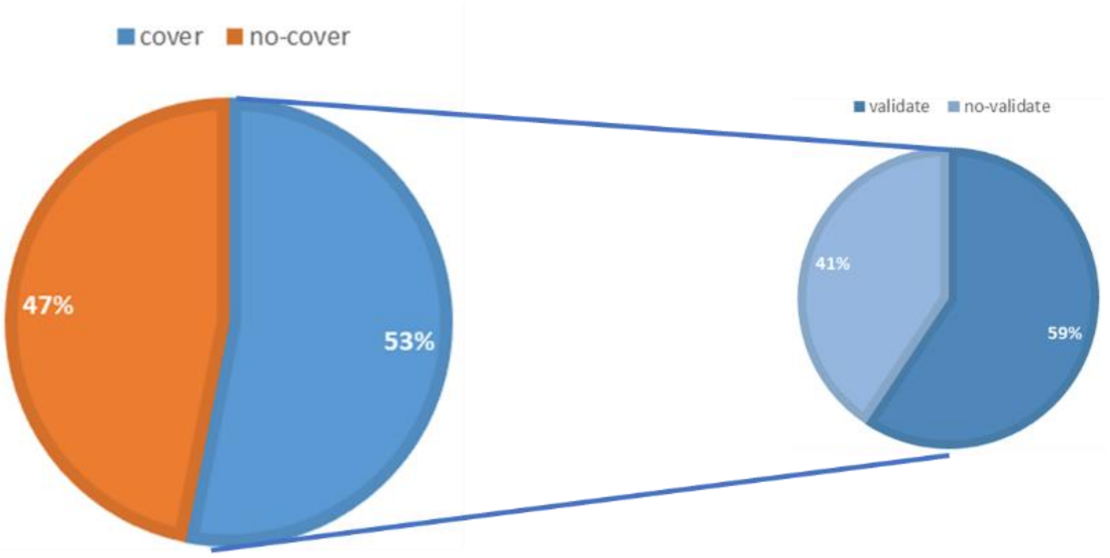

Figure S11 Validation of DAS genes identified in the muscle tissues between mule and horse by the full-length transcriptome data generated from Pac-Bio sequencing platform.

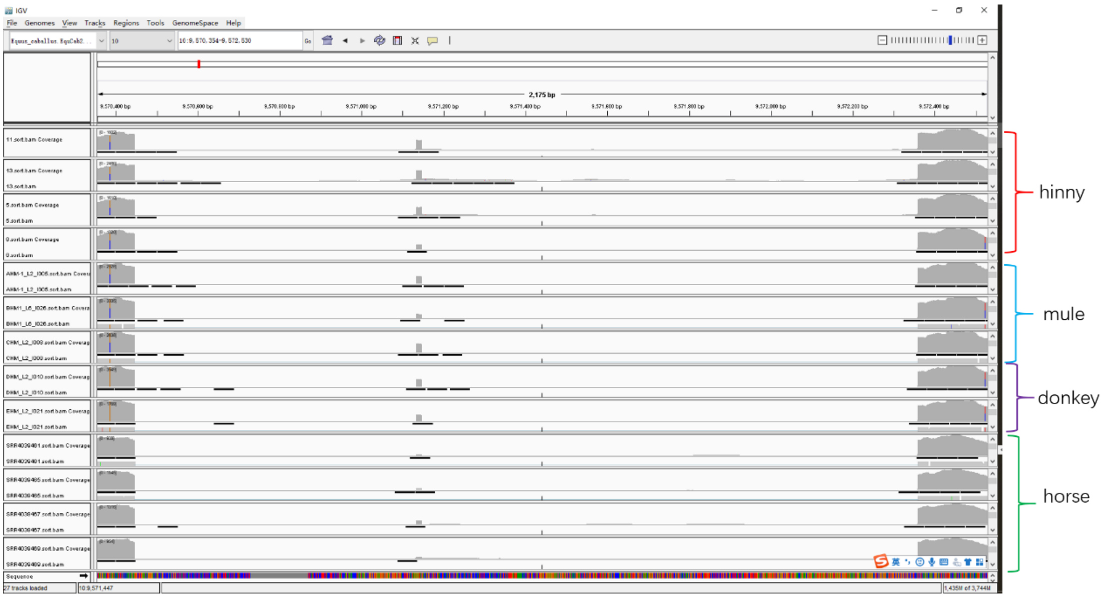

Figure S12 The result of transcriptome data mapping on the *TNNC2* gene among the muscle tissues of hinny, mule, donkey and horse.

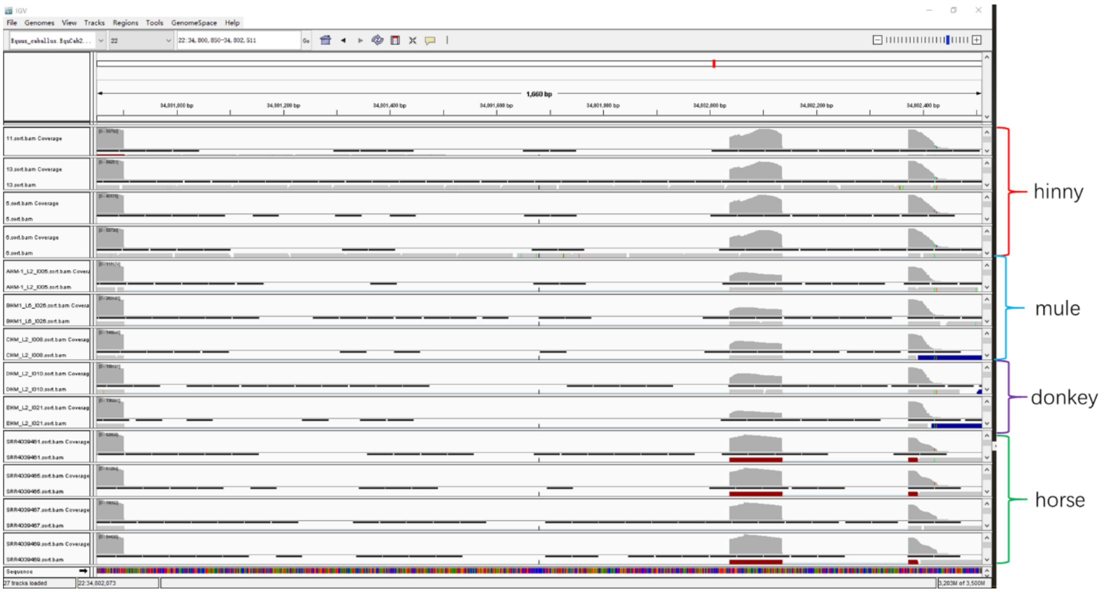

Figure S13 The result of transcriptome data mapping on the *RYR1* gene among the muscle tissues of hinny, mule, donkey and horse.
